# Molecular Characterization of *Leishmania* Species Isolated from Cutaneous Leishmaniasis in Yemen

**DOI:** 10.1371/journal.pone.0012879

**Published:** 2010-09-20

**Authors:** Mohammed A. K. Mahdy, Hesham M. Al-Mekhlafi, Abdulsalam M. Al-Mekhlafi, Yvonne A. L. Lim, Naemah O. M. Bin Shuaib, Ahmed A. Azazy, Rohela Mahmud

**Affiliations:** 1 Department of Parasitology, Faculty of Medicine, University of Malaya, Kuala Lumpur, Malaysia; 2 Central Health Laboratory, Sana'a, Yemen; 3 Department of Parasitology, Faculty of Medicine, Sana'a University, Sana'a, Yemen; London School of Hygiene and Tropical Medicine, United Kingdom

## Abstract

**Background:**

Cutaneous leishmaniasis (CL) is a neglected tropical disease endemic in the tropics and subtropics with a global yearly incidence of 1.5 million. Although CL is the most common form of leishmaniasis, which is responsible for 60% of DALYs lost due to tropical-cluster diseases prevalent in Yemen, available information is very limited.

**Methodology/Principal Findings:**

This study was conducted to determine the molecular characterization of *Leishmania* species isolated from human cutaneous lesions in Yemen. Dermal scrapes were collected and examined for *Leishmania* amastigotes using the Giemsa staining technique. Amplification of the ribosomal internal transcribed spacer 1(ITS-1) gene was carried out using nested PCR and subsequent sequencing. The sequences from *Leishmania* isolates were subjected to phylogenetic analysis using the neighbor-joining and maximum parsimony methods. The trees identified *Leishmania tropica* from 16 isolates which were represented by two sequence types.

**Conclusions/Significance:**

The predominance of the anthroponotic species (i.e. *L. tropica*) indicates the probability of anthroponotic transmission of cutaneous leishmaniasis in Yemen. These findings will help public health authorities to build an effective control strategy taking into consideration person–to-person transmission as the main dynamic of transmission of CL.

## Introduction

Leishmaniasis is a vector-borne disease caused by the genus *Leishmania* and transmitted by sandflies belonging to 20 species of the genus *Phlebotomus*. The disease is categorized as one of the “most neglected diseases” [1] and has strong and complex associations with poverty [2]. Leishmaniasis has been classified mainly into visceral leishmaniasis (VL) and cutaneous leishmaniasis (CL). As for CL, it is a skin disease endemic in the old world (Southern Europe, Middle East, Africa and central Asia) and in the Americas. In the old world, CL is caused mainly by *Leishmania tropica* [3], *Leishmania major* [4] and *Leishmania aethiopica* [5]. However, *Leishmania infantum* and *Leishmania donovani* have also been reported but are less common [6], [7], [8]. In the Americas, CL is mainly caused by *L. braziliensis* and *L. mexicana* [9].

Yemen is one of the poorest countries in the world with over 45% of the population living on less than 2 USD a day [10] and is endemic for seven neglected tropical diseases [11]. Leishmaniasis is a public health problem with a nationwide distribution and is responsible for 60% of DALYs lost due to tropical-cluster diseases prevalent in Yemen [12]. The most common form of leishmaniasis in Yemen is CL which is mutilating, disfiguring and sometimes disabling in the case of multiple lesions. The clinical pattern of the disease showed variation in the severity and duration and low response to treatment in some cases [8], [13], [14] raising the possibility of the existence of different *Leishmania* species causing CL. In addition, the current routine method using microscopic examination of Giemsa stained smears for the diagnosis of leishmaniasis does not enable species identification. The inability to determine the species of *Leishmania* limits the knowledge associated with CL. Species identification is crucial because although it is known that the common causative agent of CL is *L. tropica*, this same species has also been identified as causing VL which is the more severe type of leishmaniasis [15], [16], [17]. This study was designed to identify the causative *Leishmania* species for CL in Yemen. This information will improve our knowledge about the dynamics of disease transmission which, in turn, will assist public health authorities in establishing effective control strategies based on a comprehensive understanding of the epidemiology of leishmaniasis in Yemen.

## Materials and Methods

### Microscopic examination

The study was carried out on Giemsa stained smears, positive for *Leishmania* from the archives of the Central Health Laboratory, Sana'a, Yemen which is referral diagnostic laboratory under the Ministry of Health. Biodata and the geographical origin of patients cannot be retrieved from the records. Of the 53 patients with skin lesions suspected to be caused leishmaniasis who were referred to the Central Health Laboratory, 22 patients were positive for *Leishmania* amastigote using Giemsa stained smears and were included in the molecular analysis. The Giemsa smears were re-examined in the Department of Parasitology, Faculty of Medicine, University of Malaya, Malaysia under 1000× magnification. The smear was considered negative if amastigotes were not found after the microscopic examination of 1000 oil immersion field (OIF). Amastigote density was quantified using a semi-quantitative scale; +, 1 amastigote/whole slide to 1 amastigote/OIF in a total of at least 100 OIF; ++, 2–10 amastigotes/OIF in a total of at least 50 OIF; +++, 11–20 amastigotes/OIF in a total of at least 50 OIF; ++++, >21 amastigotes/OIF in a total of at least 10 OIF [18].

### Ethical approval

The study was approved by the Faculty of Medicine and Health Sciences, Sana'a University, Yemen. No information on the patients has been presented in this research.

### Polymerase chain reaction

DNA was extracted from Giemsa stained smears positive for *Leishmania*. The slides were first cleaned with chloroform to remove oil. Fifty µl of TE buffer was transferred onto the smear and at least half of the smear was completely wiped off the slide using Whatman 1 filter paper and transferred into 1.5 ml microcentrifuge tubes. DNA was then extracted using the Qiagen DNeasy Blood Tissue Kit (Hilden, Germany) according to the manufacturer's instructions. DNA was eluted in 100 µl and 50 µl of distilled water from slides with high and low parasitic intensity, respectively.

A Nested PCR assay was used to amplify the ribosomal internal transcribed spacer 1(ITS-1) region as described previously [19]. Primary PCR was carried out with primers LITSR (forward: 5′-CTG GAT CAT TTT CCG ATG -3′) and LITSV (reverse: 5′-ACA CTC AGG TCT GTA AAC-3′) and the secondary PCR with primers LITSR and L5.8S (reverse: 5′-TGA TAC CAC TTA TCG CAC TT-3′). Both primary and secondary PCRs were performed in a 50 µl reaction volume containing 0.5 uM of each primer (Bio Basic Inc, Canada), 2 U of *iTaq*™ *plus* DNA polymerase (iNtRON BIOTECHNOLOGY, Seoul, Korea), 1× of *iTaq*™ *plus* reaction, 200 mM of each dNTPs, and 1.5 mM MgCl_2_. Five µl of DNA template was used in both primary and secondary PCRs. In both amplifications, samples were incubated in the MyCycler thermal cycler (Bio-Rad, Hercules, USA) under the following conditions: denaturing step at 95°C for 2 min, followed by 40 cycles of denaturing for 20 s at 95°C, annealing for 30 s at 53°C and extension for 60 s at 72°C, followed by a final extension at 72°C for 6 min. The PCR products were subjected to electrophoresis on 2% agarose gels and stained with ethidium bromide.

### DNA sequencing and phylogenetic analysis

The DNA was purified using the QIAquick PCR purification Kit (QIAgen, Germany) according to the manufacturer's instructions. Cycle sequencing (bidirectional) was carried out using the ABI PRISM 1 BigDyeTM terminator v3.0 Ready Reaction Cycle Sequencing Kit (Applied Biosystems, USA) in a 3700 DNA Analyzer (Applied Biosystems, USA). The sequences representing all samples were aligned with previously published sequences as listed in Table 1, using the program MEGA4 (www.megasoftware.net). Similarity searches were carried out using the Basic Local Alignment Search Tool (BLAST) [20]. Neighbor-joining (NJ) and maximum parsimony (MP) analyses were performed in MEGA4. NJ analyses were performed with distances calculated with the Kimura 2-parameter [21]. Unweighted parsimony analyses were performed using the Close-Neighbor-Interchange algorithm [22]. To evaluate the support for inferred topologies, bootstrapping [23] was carried out using 1000 replicates.

**Table 1:** Sequences of *Leishmania* species used in the phylogenetic analysis.

Isolate	GenBank accession number*
Isolates (2, 3, 5, 6, 7, 8, 9, 10, 14, 16, and 17)	**GU561644**
Isolates (4, 11, 20, 29, and 21)	**GU561643**
*L. tropica*	FJ948456.1
*L. ethiopica*	GQ920674.1
*L. donovani*	GQ332356.1
*L. infantum*	GQ332359
*L. major*	GQ332361.1

*Accession numbers in bold represent sequences from this study.

## Results

**Figure 1:**
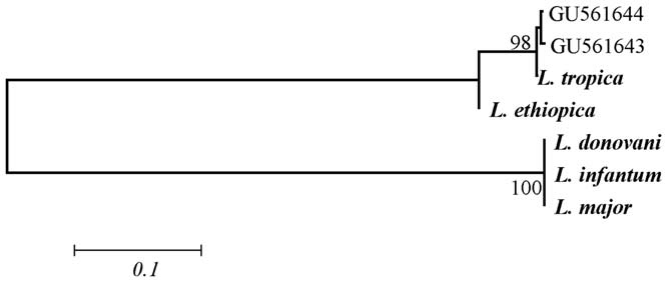
Phylogenetic analysis using Neighbor-joining Method. Neighbor-joining (NJ) tree displaying the relationships of 2 sequences (GU561643, GU561644) representing 16 Leishmania isolates. Bootstrap support of more than 90% and distance are indicated. Bold-type represents reference sequences for Leishmania species from GenBank.

**Figure 2:**
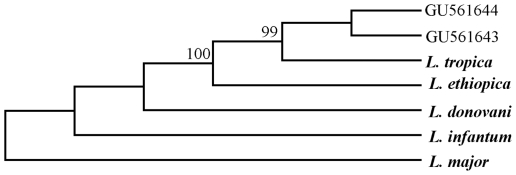
Phylogenetic analysis using Maximum parsimony Method. Maximum parsimony (MP) tree displaying the relationships of 2 sequences (GU561643, GU561644) representing 16 Leishmania isolates. Bootstrap support of more than 90% is indicated. Bold-type represents reference sequences for Leishmania species from GenBank.

Microscopic examination detected *Leishmania* amastigotes in 22 smears of the 53 smears collected from patients with suspected cutaneous leishmaniasis who attended the Central Health Laboratory. Amastigote density was ++++, +++, ++ and + in 27%, 14%, 14% and 45% of the samples, respectively. Of the 22 microscopically positive smears for *Leishmania* species, amplicons (**∼**350 bp) were produced from 17 samples (77%). Sixteen of these amplicons were successfully sequenced in both directions except one. Sequence analysis divided the samples into two groups; group1 contained eleven isolates (2, 3, 5, 6, 7, 8, 9, 10, 14, 16, and 17) and had identical sequences and group 2 consisted of five isolates (4, 11, 20, 29, and 21) and had identical sequences. Polymorphism between the two sequence types was detected in 4 positions (4 SNP). For phylogenetic analysis, two sequence types representing all 16 isolates and five reference sequences obtained from GenBank representing *L. donovani*, *L. infantum*, *L. major*, *L. aethiopica* and *L. tropica* (table 1) were multiple aligned. The inferred phylogenetic trees based on Neighbor-Joining (Figure 1) and Maximum Parsimony (Figure 2) were concordant in topology with strong support. In the NJ method, *Leishmania* isolates were grouped with *L. tropica* in one cluster (98% bootstrap), while the MP method placed *Leishmania* isolates in one clade with *L. tropica* with 99% bootstrap support.

## Discussion

Using molecular characterization, this present study identified that archived Giemsa stained smears found positive for cutaneous leishmaniasis were caused by *L. tropica*. *L. tropica* as a causative agent for CL was previously reported in Tihama coastal plain, Yemen in 1989 [24]. Almost two decades later, Khatri and his colleagues detected *Leishmania* amastigotes in 128 cases of CL from northern Yemen. Of these, four CL cases were characterized using the isoenzyme electrophoresis technique and *L. tropica* was identified in all these cases [14]. In their study, 49% of skin smears were highly positive compared to 27% of CL cases in our study.

However, *L. tropica* was not the only species identified to be the causative agent of CL. In 1993, *L. donovani* was isolated from a cutaneous lesion in a tourist from southern France who visited Yemen for two weeks in 1992 [25]. He was reported to have been bitten repeatedly by sandflies. The patient did not present any signs and symptoms of VL although *L. donovani* is more commonly associated with VL. Again, a recent study carried out using PCR-RFLP involving 155 CL cases originated from 10 governorates of northern Yemen highlighted the possibility of CL being caused by more than one species of *Leishmania*. This study detected *L. tropica* in 133 cases (85.80%), *L. infantum* in 17 cases (10.97%) and *L. donovani* in 5 cases (3.23%) [8]. Nonetheless, based on findings from these studies in Yemen, *L. tropica* is still the predominant species responsible for CL. The predominance of *L. tropica* as a causative agent of CL has been reported from Saudi Arabia [26].

Although *L. tropica* most commonly causes CL, it has been isolated from VL cases [27], [28] The isolation of *L. tropica* from human cases of VL among the US soldiers returning from Operation Desert Storm in the Gulf countries [15], [16], [29] raised a question as to whether *L. tropica* could manifest in the form of VL in Yemen. The potential of this species to cause VL, the most virulent form of leishmaniasis, will exacerbate the situation in Yemen since this form of the disease may respond poorly to different therapeutic regimens [8].

*L. tropica* is commonly stated to be anthroponotic [30], although zoonotic transmission has been reported from Greece [31], Kenya [32], Jordan [33] and Saudi Arabia [26]. In Yemen, a survey of reservoir hosts has not been performed to date based on the assumption that CL is usually an anthroponotic infection. Moreover, not much is known about the vector of *L. tropica*. However, *Phlebotomus sergenti*, the most common vector of *L. tropica* [30], was detected in an entomological collection from Taiz governorate in 1951 [34].

Leishmaniasis is a neglected disease in Yemen. The true incidence is not well reflected as only a few published documents are available. The disease burden is underestimated as there is no national reporting system in place and no active case detection is being carried out. Furthermore, there are no systematic national efforts to control the disease. Since there is a scarcity of research done on *Leishmania* in Yemen, further investigations on aspects of the type of vectors, the vectors' behaviour and feeding preference, insecticide susceptibility, the presence of reservoir hosts, risk factors of acquiring the infection and drug resistance are crucial for effective control strategies to be formulated.

This study is the first study in Yemen to identify the *Leishmania* species causing CL based on sequencing the ITS locus. Previous research carried out by Khatri et al. [8] was based on PCR-RFLP. Building a GenBank database on *Leishmania* in Yemen could be useful for future comparison studies. However, findings from the current study are limited by the small sample size and the lack of the geographical information on *Leishmania* cases.

The findings of this present study and the previous studies conclude that CL is most commonly caused by *L. tropica* suggesting the anthroponotic transmission of CL in Yemen. Leishmaniasis is a neglected disease and does not receive much attention from public health authorities. An effective strategy for the control of leishmaniaisis should be developed, embedded in the national health plans and harmonized with operational, entomological and epidemiological research to ensure its continuing effectiveness. An effective surveillance system is imperative to monitor cases and improve disease awareness among medical personnel.
